# Mapping the Flowering of an Invasive Plant Using Unmanned Aerial Vehicles: Is There Potential for Biocontrol Monitoring?

**DOI:** 10.3389/fpls.2018.00293

**Published:** 2018-03-08

**Authors:** Nuno C. de Sá, Paula Castro, Sabrina Carvalho, Elizabete Marchante, Francisco A. López-Núñez, Hélia Marchante

**Affiliations:** ^1^Centre for Functional Ecology, Department of Life Sciences, University of Coimbra, Coimbra, Portugal; ^2^CoolFarm S.A., Instituto Pedro Nunes, Coimbra, Portugal; ^3^Instituto Politécnico de Coimbra, Escola Superior Agrária de Coimbra, Coimbra, Portugal

**Keywords:** invasive plant species, Remote Sensing, unmanned aerial vehicles, coastal habitats, biocontrol agent monitoring, *Acacia longifolia*, flower mapping

## Abstract

Invasion by alien species is a worldwide phenomenon with negative consequences at both natural and production areas. *Acacia longifolia* is an invasive shrub/small tree well known for its negative ecological impacts in several places around the world. The recent introduction of a biocontrol agent (*Trichilogaster acaciaelongifoliae*), an Australian bud-galling wasp which decreases flowering of *A. longifolia*, in Portugal, demands the development of a cost-efficient method to monitor its establishment. We tested how unmanned aerial vehicles (UAV) can be used to map *A. longifolia* flowering. Our core assumption is as the population of the biocontrol agent increases, its impacts on the reduction of *A. longifolia* flowering will be increasingly visible. Additionally, we tested if there is a simple linear correlation between the number of flowers of *A. longifolia* counted in field and the area covered by flowers in the UAV imagery. UAV imagery was acquired over seven coastal areas including frontal dunes, interior sand dunes and pine forests considering two phenological stages: peak and off-peak flowering season. The number of flowers of *A. longifolia* was counted, in a minimum of 60 1 m^2^ quadrats per study area. For each study area, flower presence/absence maps were obtained using supervised Random Forest. The correlation between the number of flowers and the area covered by flowering plants could then be tested. The flowering of *A. longifolia* was mapped using UAV mounted with RGB and CIR Cannon IXUS/ELPH cameras (Overall Accuracy > 0.96; Cohen’s Kappa > 0.85) varying according to habitat type and flowering season. The correlation between the number of flowers counted and the area covered by flowering was weak (*r^2^* between 0.0134 and 0.156). This is probably explained, at least partially, by the high variability of *A. longifolia* in what regards flowering morphology and distribution. The very high accuracy of our approach to map *A. longifolia* flowering proved to be cost efficient and replicable, showing great potential for detecting the future decrease in flowering promoted by the biocontrol agent. The attempt to provide a low-cost method to estimate *A. longifolia* flower productivity using UAV failed, but it provided valuable insights on the future steps.

## Introduction

Biological invasions are one of the main factors of habitat changes, resulting in immeasurable losses to economy and biodiversity (Hulme, 2009; Perrings et al., 2010). Often, long-term invasions trigger drastic changes in ecosystem functioning, in land cover and in biotic communities both above and below ground (Marchante et al., 2008; Hellmann et al., 2017). Such changes may be particularly aggravated when native and invasive plants have different life forms (Marchante et al., 2015).

The European Commission has recently recognized the severity of the problems caused by Invasive Alien Species through the Regulation (EU) No 1143/2014 of the European Parliament and the Council of the European Union, clearly stressing (amongst other strategies) the importance of effective management practices (European Union, 2014). Methods currently used in Europe to control invasive plants are frequently prohibitively expensive and often unsuccessful, unsustainable, and/or environmentally damaging. These, associated with the increasing pressure to reduce the use of herbicides stresses that it is crucial to find more sustainable, inexpensive, and environmentally friendly control approaches.

*Acacia longifolia* (Andrews) Willd. (Sydney Golden Wattle; *Fabaceae*) is one of several Australian *Acacia* species that are invasive in many regions around the world (Rejmánek and Richardson, 2013) including in southern Europe where it has proliferated mostly on coastal areas (Marchante et al., 2014; Cesar de Sa et al., 2017). This leguminous shrub/small tree has a long track of diverse ecological impacts in coastal systems (Marchante et al., 2003, 2008), where it replaces native herbaceous and shrub dune communities and proliferates under maritime pine forests. The long-term invasion by *A. longifolia* has significant impacts on soil properties, altering its biological and chemical composition (Marchante et al., 2009; Hellmann et al., 2017) and hindering the recovery of invaded habitats (Marchante et al., 2011).

The intentional introduction of natural enemies to control invasive plants is frequently considered as a sustainable and environmentally friendly methodology around the world (Shaw et al., 2016). It has been used for over a century worldwide (Murphy and Evans, 2009), with nearly 550 agents released against over 224 plant species in 130 countries (Winston et al., 2014). Many introductions of biocontrol agents resulted in either complete (ca. 1/3 of total cases of invasive plants) or moderate (higher number of agents) levels of success (Murphy and Evans, 2009). Despite this, biological control can be considered as potentially dangerous, current practice assures that before a species is cleared for release a full range of tests and risk analyses are performed to assure that the risk is minimal. Yet, in Europe, several reasons including (amongst others) an apparent ignorance of the potential of biological control of weeds amid policy makers and some level of risk aversion (Sheppard and Shaw, 2006) has delayed the use of biocontrol against invasive plants until 2010. After that, it has been used three times: two in the UK, with releases in 2010 and 2014 (Shaw et al., 2011; Tanner et al., 2015), and the third in Portugal with the first release of *Trichilogaster acaciaelongifoliae* (Froggatt) (*Hymenoptera: Pteromalidae*), to control *A. longifolia*, in late 2015 (Marchante et al., 2017). It is expected that the biocontrol agent (BCA) *T. acaciaelongifoliae* (an Australian bud galling wasp) will significantly reduce the flower production of *A. longifolia* (Dennill, 1985; Dennill et al., 1999), thus disrupting its capacity to renew the seed bank (Marchante et al., 2010).

The introduction of *T. acaciaelongifoliae* began in November 2015 with its release in eight sites along the Portuguese coast (López-Núñez et al., 2017; Marchante et al., 2017). To ensure the success of this biocontrol measure it important to monitor its establishment and impact on *A. longifolia*. However, the fieldwork is time-consuming, expensive, and each field campaign is only capable of covering small areas. Furthermore, as the BCA population increases and spreads, it will be virtually impossible to monitor its extent only through field sampling. Unmanned Aerial Vehicles (UAV) can potentially offer a cost-effective solution to address this challenge. Our assumption is that if it is possible to map the flowering of *A. longifolia* using low-cost UAV platforms equipped with “off-the-shelf” digital camera sensors, then it is possible to monitor the establishment of the BCA at low cost as well. Ultimately, we expect that monitoring the loss of flowering can be used to continuously monitor the establishment of the BCA. The use of remotely sensed data has rapidly increased and has nowadays a wide range of applications in many different fields, with potential to become a key tool for ecological research and conservation (Horning et al., 2010; Gonzalez et al., 2016) including for the study of invasive alien plants (Cesar de Sa et al., 2017; Weisberg et al., 2017).

Unmanned aerial vehicles offer unique opportunities for Spatial Ecology because of their ability to almost on-demand acquire very high-resolution imagery (Anderson and Gaston, 2013) and by using sensors tailored to the task as well as 3D structure of plants which will allow further inferences of plants physiological and structural traits at an unprecedented spatial scale (Niphadkar and Nagendra, 2016). Remote Sensing of invasive alien plants is often difficult because these occur in disturbed areas, “mixed pixels” and under canopy (Bradley, 2014). UAVs represent an exceptional opportunity to detect invasive plants due to their operational flexibility (Müllerová et al., 2017a,b). This allows researchers and stakeholders to improve their mapping efforts by focusing on a particular phenological stage of the target species (Hill et al., 2016), e.g., the peak and post peak of flowering season.

Attempts to use plant phenology to improve detection by Remote Sensing are not new (Everitt et al., 1995; Gorsevski, 2013; Khwarahm et al., 2017) but efforts like these are often hindered by lack of easily accessible and low cost data. The impact of flowering in the spectral characteristics of plants can sometimes lead to increased confusion between classes (He et al., 2011) which implies a need for deeper characterization (Andrew and Ustin, 2006). While *A. longifolia* leaf spectral responses have been thoroughly measured (Lehmann et al., 2015a; Große-Stoltenberg et al., 2016) there is lack of similar data collected on its flowering. Mapping *A. longifolia* using airborne hyperspectral sensors has been shown to be successful (Pandey et al., 2014) but these methods are still too costly to be included in a continuous monitoring effort.

In Portugal, *A. longifolia* flowering period usually peaks during winter (mainly between late December and March) (Morais and Freitas, 2015) before most other co-occurring plants begin to flower. During these months, areas invaded by *A. longifolia* are clearly visible due to their bright yellow flowering bloom. We hypothesize that the high resolution of the Remote Sensing equipment mounted in UAVs can capture this species flowering features, rarely captured via satellite due to winter cloud cover. By testing the potential for mapping the flowering of *A. longifolia* we additionally aim to demonstrate its potential for monitor the future impact of the BCA. This may become a novel approach for monitoring the impact of insects on the phenological cycle of invasive plant species, ensuring low cost and repeatability.

Previous works have shown a linear relationship between the area covered by flowers in photographs captured by digital cameras and the number of flowers of *Lesquerella fendleri* (Gray) S. Wats (an herbaceous species with regular distribution of flowers) grown in controlled experimental conditions (Adamsen et al., 2000; Thorp and Dierig, 2011). Based on this, our second aim is to test if such relationship exists when the plant is a woody species, *A. longifolia*, with variable distribution of flowers, occurring in the wild and using UAV imagery. This would allow making estimations of numbers of flower (or other morphological parameter) through UAV imagery.

Summarizing, the objectives of this work were to evaluate the use of UAV Remote Sensing imagery to: (a) detect and map the flowering of the invasive plant *A. longifolia*, and (b) test if there is a linear relationship between the flower cover detected by the UAV and the number of flowers of *A. longifolia* measured in field. This is particularly important as this method is intended to be applied in the future to detect the effects of the BCA on the density of flowers, which is expected to decrease substantially in the future (Dennill et al., 1999).

## Materials and Methods

### Study Species and System

**FIGURE 1:**
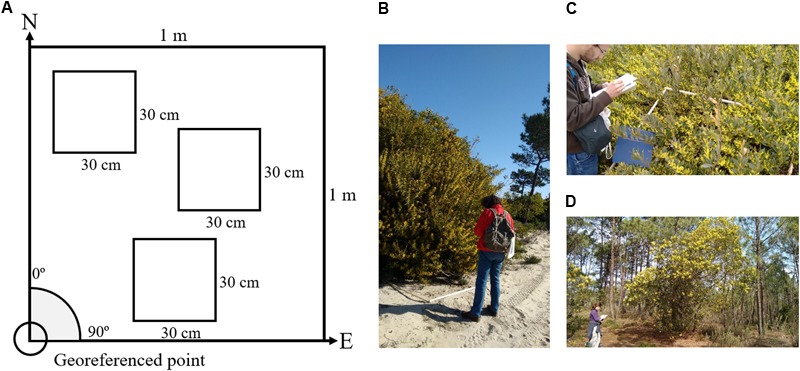
Scheme of how the flower counting was performed in the field and examples of field data collection by the team. **(A)** The bottom left corner was georeferenced and the quadrat had one side oriented toward N and the other oriented toward east. **(B–D)** Show examples of data being collected in the field with the 1 m^2^ quadrat. The variability of the flowering of this species is visible: **(B)** Sample collected in the interior dunes where the flowering extends throughout the whole branches of the plant. **(C)** In the frontal dunes where plants are smaller and flowering does not extend to the end of the branch and is instead “hidden” below the canopy of the plant. **(D)** Is a typical example of how *A. longifolia* morphologically adapts to the pine forest by growing in height as opposed to width and shows a decrease in flower productivity.

*Acacia longifolia* (Andrews) Willd. is easily identifiable both during the flowering season due to the bloom of yellow flowers as well as during the fruitification when its seed pods begin to brown and remain on the branches. It can develop as a shrub (often up to 4 m in height), when occurring in primary dunes, and as small tree (up to 8 m in height) when occurring more inland, also below the canopy of other species (e.g., maritime pines) (Cesar de Sa et al., 2017; Invasoras, 2017). During fieldwork we observed a high variability in flower patterns amongst plants depending on its morphology (tree or shrub) and on the position in the dune system (**Figures 1B,C**). For example, while *A. longifolia* generally produces flowers throughout the broad terminal part of the branches (**Figure 1B**), it occasionally produces less flowers in the tip of the branches when in the primary dunes (**Figure 1C**); and produces much lesser amount when under pine canopies and often closer to the tip of the branch (**Figure 1D**).

**FIGURE 2:**
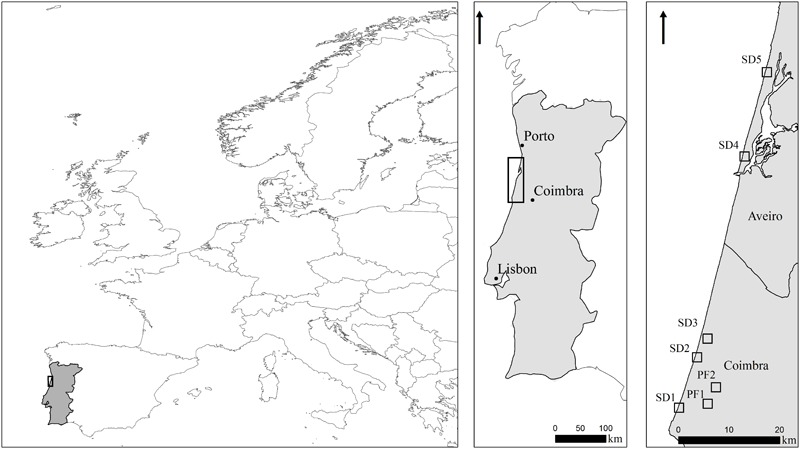
Study sites location. SD1, SD2, Quiaios sand dunes; SD3, Tocha secondary sand dunes; SD4, São Jacinto sand dunes; SD5, Torreira sand dunes. PF1, PF2, Quiaios pine forests.

This study was carried out on seven sites distributed along ca. 80 km of the central-northern coast of Portugal (**Figure 2** and **Table 1**). Sites were selected in order to include the most common coastal habitats invaded by *A. longifolia*: (1) several plant communities in sand dunes (SD), including primary dunes, interdunes and secondary dunes (Cesar de Sa et al., 2017); maritime pine occurs in these areas but generally does not dominate (SD1, SD2, SD3, SD4, SD5); and (2) pine forests plantations (hereafter pine forests, PF), where maritime pine dominates and *A. longifolia* occurs in the understory (PF1, PF2).

**Table 1:** Data acquisition summary: characterization of sites, UAV flights, and fieldwork sampling done to quantify the number of flowers.

	Site characteristics		Image acquisition			Fieldwork	
Site	Flowering status	Habitat	Date	Time of day	Resolution (cm)	Flower sampling	Quadrats sampled
SD1	Peak	Primary dune	10/03/2015	Morning	4	11/03/2015	71
SD2	Peak	Primary dune	11/03/2015	Morning	4	12/03/2015	71
SD3	Peak	Secondary dune	11/03/2015	Afternoon	4	13/03/2015	63
SD4	Post peak	Primary and interdune	04/04/2015	Morning	6	25/03/2015	65
SD5	Post peak	Primary dune	04/04/2015	Afternoon	6	06/04/2014	60
PF1	Peak	Pine forest	10/03/2015	Afternoon	6	–	0
PF2	Peak	Pine forest	13/03/2015	Afternoon	5	18/03/2015	78

*Due to logistics and technical difficulties it was not possible to perform fieldwork in PF1. Since sampling locations were randomly selected not all areas of 50 × 50 m had *A. longifolia*.*

The dunes of Quiaios (SD1 and SD2, **Figure 2**) are a coastal strain of primary and secondary sand dunes with associated pine forests that extends inland. These sites are characterized by a mosaic of several well-established plant communities, including native perennial herbs (e.g., *Ammophila arenaria* and *Corynephorus canescens*) alternating with chamaephytic shrublands (e.g., *Corema album, Cistus salviifolius, Artemisia campestris*). Between the different plants assemblages there are large areas dominated by invasive plant species (e.g., *Carpobrotus edulis, Arundo donax, Acacia saligna*, and *A. longifolia*).

Tocha secondary sand dunes (SD3, **Figure 2**) are characterized by a singular sand dune string conformation, perpendicular to the shore line. Like Quiaios dunes (SD1 and SD2) it is mainly dominated by diverse well adapted littoral plant communities (with *A. arenaria, C. album* and *A. campestris* as the most common species) alternating with areas dominated by alien plant species, such as *C. edulis* and *A. longifolia*. SD3 burned in 1993 which triggered the expansion of *A. longifolia*, leading to the replacement of maritime pine stands by monospecific stands of *A. longifolia*. São Jacinto (SD4) and Torreira (SD5) sand dunes are located further north and are characterized by primary dunes highly invaded by *A. longifolia*. SD4 shows maritime pines and large patches of invasion by *A. longifolia.* In SD5 the maritime pines co-occur with secondary dune communities and are also mixed with invasive *Acacia dealbata* and *Acacia melanoxylon*. On the other hand, the pine forests of Quiaios (PF1 and PF2, **Figure 2**) include sparse plantations of *Pinus pinaster* with scattered patches of *Halimium halimifolium* and *C. salviifolius*. PF1 also has *Eucalyptus globulus* plantations. Both PF1 and PF2 show a varying degree of invasion by different *Acacia* species, including *A. longifolia*.

### Fieldwork and UAV Data Acquisition

The field work was divided in two distinct main tasks performed as closely as possible in time (**Table 1**): (1) remote imagery acquisition by low cost digital cameras mounted on UAV, and (2) fieldwork sampling to quantify the number of spikes (hereafter referred as flowers) of *A. longifolia* produced per m^2^ in randomly selected areas within each site.

Both field sampling and the UAV flights were performed in two seasons: (1) during flowering peak and (2) at the end of the flowering season of *A. longifolia* (**Table 1**), in order to test the accuracy in detecting flowers in both periods. By studying different phenological stages and habitats we expected to obtain a broad overview of the possibilities and limitations of this method.

Unmanned aerial vehicles flights were executed by Firemap SA^1^, between March and April 2015, using the senseFly eBee^2^ mounted, alternately, with one of two versions [regular RGB or filtered Colour-infrared (CIR)] of the Cannon IXUS/ ELPH, 16.0 MP, 6.16 mm × 4.62 mm sensor. Since two different cameras were operated with the same UAV, it was necessary to perform two flights over the same area and therefore the RGB and CIR acquisition had on average 1 h of difference between them. All flights were performed between 100 and 160 m above ground altitude depending on the meteorological and local terrain conditions and this resulted in different resolution of the final products (**Table 1**).

All the UAV data was pre-processed and delivered by Firemap SA using Structure-from-motion (SFM) available in Agisoft Photoscan version 1.2.0. Besides delivering the orthophotos of each location with 4 bands (RGB and NIR), the SFM by-products of Digital Surface Model (DSM) and Digital Elevation Model (DEM) were also delivered. The DEM was obtained by cloud-point semi-supervised classification methods which are readily available in Agisoft Photoscan. The Canopy Height Model (CHM) was obtained by subtracting the DEM from the DSM (Matese et al., 2017). This resulted in 5 different covariates used for the classification exercise: Red, Blue, Green, and NIR bands and the CHM.

Because the flights were performed between March and April (*A. longifolia* flowered very late in the Portuguese winter of 2015) and often near the coastline, it was not possible to deploy the UAV in all locations during an ideal timeframe (**Table 1**). The flights in each site covered approximately an area of 65 ha. Also, per area, a minimum of nine well spread-out ground control points were collected with sub centimeter accuracy using a Trimble GPS XH 6000 antenna receiver to assist in the processing of the final products (Benassi et al., 2017) as well as for alignment of the RGB and CIR acquisitions.

In each site, 30 areas of 50 m × 50 m were randomly selected for further field sampling which meant not all the pre-selected areas would have *A. longifolia*. Within each area with *A. longifolia* presence, a minimum of three 1 m^2^ quadrats were sampled for flowering counts (**Figure 1** and **Table 1**). A minimum of 60 1 m^2^ quadrat samples were collected in each study area (**Table 1**). Due to the extremely high number of flowers present in 1 m^2^, it was necessary to adapt the method of flower counting which would otherwise become unfeasible. So, within each quadrat, the flowers were counted in (at least) 3 smaller subplots of 30 cm × 30 cm (**Figure 1A**). Also, only the number of flowers in the first 30 cm starting from the tip of the branch were considered. These subplots were chosen in order to be representative of the flowering within that 1 m^2^ quadrat (**Figure 1**). The smaller 30 cm × 30 cm subplots were then extrapolated to number of flower per m^2^. This method was adapted from the methodology used by Morais and Freitas (2015) to provide an estimate of the number of flowers on a sampled area m^2^. In total, data on 408 m^2^ were collected. Each quadrat was georeferenced in the SW corner and oriented toward NE. A Trimble GeoExplorer 6000^®^ with post-processing differential correction was used to georeference the quadrat position to correctly match the field flower count sampling with the UAV observation.

### Mapping *Acacia longifolia* Flowering

To map the flower presence/absence in each image we used the Random Forest algorithm (Breiman, 2001). The Random Forest is a classification algorithm widely used in the field of Remote Sensing (Feng et al., 2015; Belgiu and Drăgut, 2016). It’s a non-parametric supervised classification which consists in an ensemble of Classification and Regression Tree’s to identify the target classes (Breiman et al., 1984; Breiman, 2001). The tree creation process consists in subsetting the training samples with replacement and using part of the data to train the tree while the remaining data is used to measure the prediction error (Belgiu and Drăgut, 2016). The final error estimate is known as the Out-of-Bag error (OOB) and is given by averaging the prediction error (Breiman, 2001; Belgiu and Drăgut, 2016).

**FIGURE 3:**
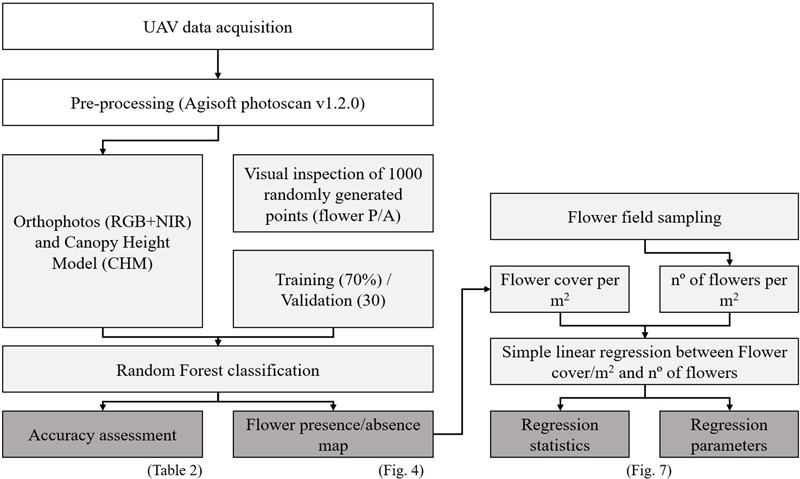
Flowchart of the process developed to evaluate both flower cover and flower count of *A. longifolia* per m^2^. The simple linear regression was performed by using the flower cover per m^2^ detected by UAV in each of the sampled quadrats.

To calibrate and validate the classification, 1000 points were randomly generated for each study area and visually identified as having flower presence or not (**Figure 3**). This is a common practice in Remote Sensing when very high resolution images are available and the distinction between the classes is clear (Feng et al., 2015; Lehmann et al., 2017; Müllerová et al., 2017b) as it is in our case (*A. longifolia* flowers vs. everything else, **Supplementary Figure S9**).

These 1000 points were then randomly separated into two groups, one used for calibration (70% of the points) and the remaining 30% used for validation which allowed assessing the Overall Accuracy and Cohen’s Kappa for each image (Lehmann et al., 2017; Müllerová et al., 2017b). To obtain the maps of *A. longifolia* flower cover/m^2^ we applied a focal filter to the binary map where the window size varied according to the image resolution (**Table 1**), effectively resampling each image to 1 m^2^.

The visual identification of the calibration/validation points was performed using ArcGIS^®^10.2 while R^®^ 3.2.1 was used for: (i) data handling raster (Hijmans and van Etten, 2012), (ii) maptools (Bivand and Lewin-Koh, 2012), (iii) Random Forest classification (Liaw and Wiener, 2002) and (iv) Presence/Absence classification accuracy tests (Freeman and Moisen, 2008).

As we aim to evaluate the feasibility of using UAV imagery to monitor the expansion of *T. acaciaelongifoliae* (specifically through its effects on the flower cover of *A. longifolia*) we analyzed the statistical distributions of the flower cover per m^2^ in each site.

### Testing Correlation Between Flower Cover by UAV and Field Measured Flower Number

In our study we followed the same approach used by Thorp and Dierig (2011). In controlled conditions these authors collected digital photographs of a small patch (0.125 m^2^) of *Lesquerella fendleri* (Gray) S. Wats and established a linear relationship between the area occupied by flowers in the photograph and the number of flowers present in the patch. We tested this method in the field for *A. longifolia* and by using UAV imagery. *Acacia longifolia* is structurally more complex than *L. fendleri*; as previously mentioned, it is a shrub/small tree which produces flowers in different heights while the latter is an herb.

To adapt the Thorp and Dierig (2011) method to imagery acquired by UAV we used the 1 m^2^ quadrats positioned in the field (as shown in **Figure 1**) to guide the extraction of the flower cover in that same location. This allowed the establishment of the linear regression between flower cover and number of flowers counted in the field. If this linear relationship between area of flower cover and number of flowers counted in the field is successfully established, then this method would allow mapping the number of flowers of *A. longifolia* using UAVs using only the area covered by flowers.

## Results

### Mapping *Acacia longifolia* Flowering

The Random Forest classification obtained very high accuracy when detecting presence of flowers of *A. longifolia* (**Table 2**). The Overall Accuracy and Cohen’s Kappa values were always higher than 0.95 and 0.85, respectively, which indicates that the color of the flowers was very well detected by UAV. The lowest Kappa values corresponded to the sites PF1 and PF2 where *A. longifolia* invades as an understory species and the flower visibility was hindered both by total canopy cover as well as shadows. Considering the imagery acquired during the peak of flowering on dune systems (SD1, SD2, and SD3), SD2 and SD3 showed the highest Kappa values of all, while the OOB and the Overall Accuracy were very high for all locations. Blue band was the most important variable for all study areas except for SD2 (**Supplementary Figure S10**) which can be indicative of the contribution the yellow flowering to the classification accuracy as yellow is a result of low blue value and a combination of red and green (Sulik and Long, 2016). In opposition, the Near-Infrared band was the least important variable for all study areas except for P01 (**Supplementary Figure S10**) which can be indicative that this band is not helpful to distinguish yellow flowers from the background.

**Table 2:** Accuracy values obtained for the Random Forest classification algorithm, for each study site.

Site	OOB (%)	1-OOB	Overall accuracy	Cohen’s kappa
SD1	3.29	0.967	0.957	0.910
SD2	2.43	0.978	0.980	0.950
SD3	2.71	0.973	0.993	0.986
SD4	3.71	0.963	0.973	0.939
SD5	3.57	0.964	0.967	0.896
PF1	0.71	0.993	0.980	0.859
PF2	1.72	0.983	0.973	0.870

**FIGURE 4:**
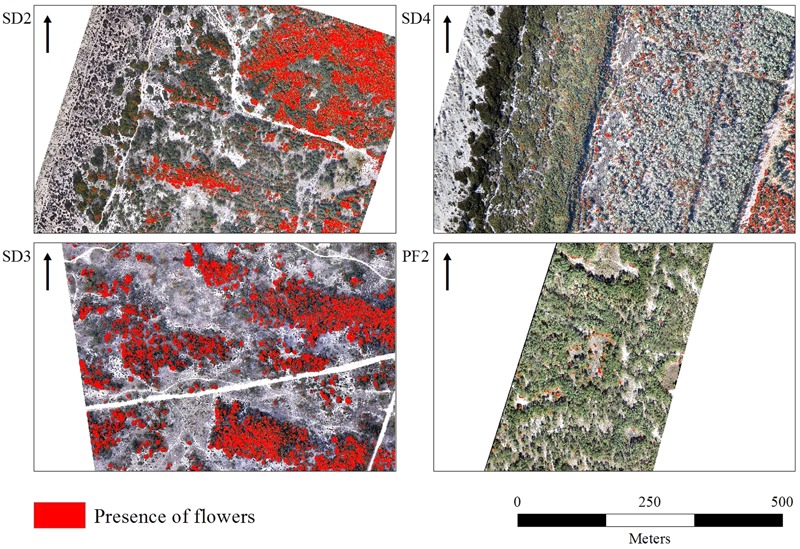
Spatial distribution of *A. longifolia* flowers detected by UAV imagery (binary maps) in selected sites representing the different situations. SD2 and SD3, peak of the flowering season; SD4, post-peak season; PF2, *A. longifolia* in the understory of the pine forest. Flower distribution in all sites may be observed on the **Supplementary Figures S1–S4**. Name of study sites as in **Figure 2**.

Clear flowering distribution patterns of *A. longifolia* were visible in images of all sites, showing specifically more presence of flowering in the dunes perpendicular to the shoreline (SD2 and SD3) (**Figure 4**) while SD1 did not have these types of dunes and also had more areas with pines. For the imagery acquired after the peak of flowering (SD4, SD5) the accuracy was lower than that of the best scenarios (0.973; 0.939 and 0.967; 0.896 respectively – **Table 2**). The flowering in these locations was harder to identify even during the extraction of the calibration/validation datasets (**Figure 4** and **Supplementary Figures S3, S4**). While still within the flowering peak, it was possible to detect flowers on the edges of open areas of the pine forests (for example PF2 in **Figure 4** and PF1 **Supplementary Figure S4**).

**FIGURE 5:**
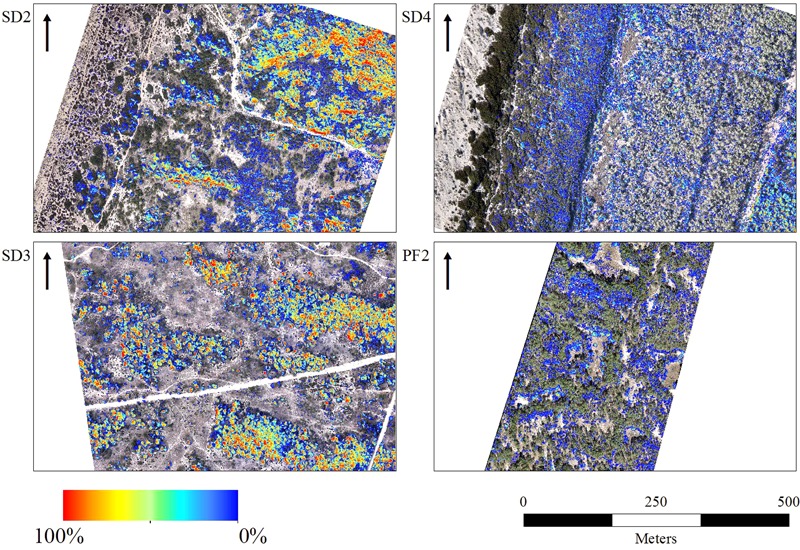
*Acacia longifolia* flower cover/m^2^ in selected sites representing the different situations. SD2 and SD3, peak of the flowering season; SD4, post-peak season; PF2, *A. longifolia* in the understory of the pine forest. Areas fully covered with flowers correspond to red; areas with only a few flowers are showed in blue. Flower cover/m^2^ in all sites may be observed on the **Supplementary Figures S5–S8**. Name of study sites as in **Figure 2**.

The flower cover maps, i.e., the total area of flower per m^2^, showed that dunes perpendicular to the shoreline (SD2 and SD3) did have more flower cover (**Figure 5**). The post peak imagery (SD4 and SD5 **Supplementary Figure S7**) showed less flowering intensity throughout the whole image (although very high rates of *A. longifolia* are known to be present), with more cover observed in the East of the study area and farther from the coastline. In pine forests (PF2) higher cover of flowers were mostly visible in the open spaces where pine canopy did not cover *A. longifolia* (**Figure 5**) but also still visible through the pine tree canopy.

**FIGURE 6:**
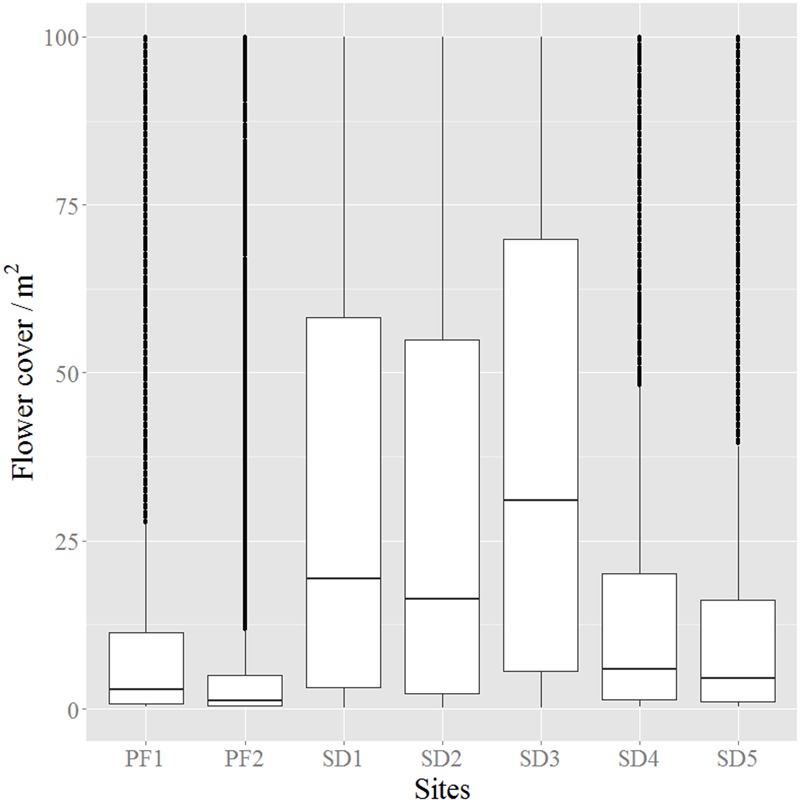
Boxplot of the pixel distribution for the flower cover/m^2^ in all study sites. Boxes represent the interquartile range from the mean; dotted points (sometimes having the appearance of bold lines) are outliers while the continuous thin line represents the maximum and minimum of the distribution. Name of study sites as in **Figure 2**.

The analysis of the distributions of flower cover per m^2^ in each site showed that all distributions were skewed, i.e., had an unbalanced distribution. In sites where flower detection was difficult, either because flowers were covered by pine canopy (PF1, PF2) or because images were acquired after the peak of flowering (SD4, SD5), the distribution was skewed toward the origin, reflecting lower values of flower cover per m^2^ with less variability (**Figure 6**). On the other hand, imagery obtained during the flowering peak period (SD1, SD2, SD3) showed not only higher average values of flower cover but also the highest variability which translates the high spatial variability of flowering patterns of the species (**Figure 6** and **Supplementary Figures S5–S8**).

### Testing Correlation Between Flower Cover by UAV and Field Measured Flower Number

**FIGURE 7:**
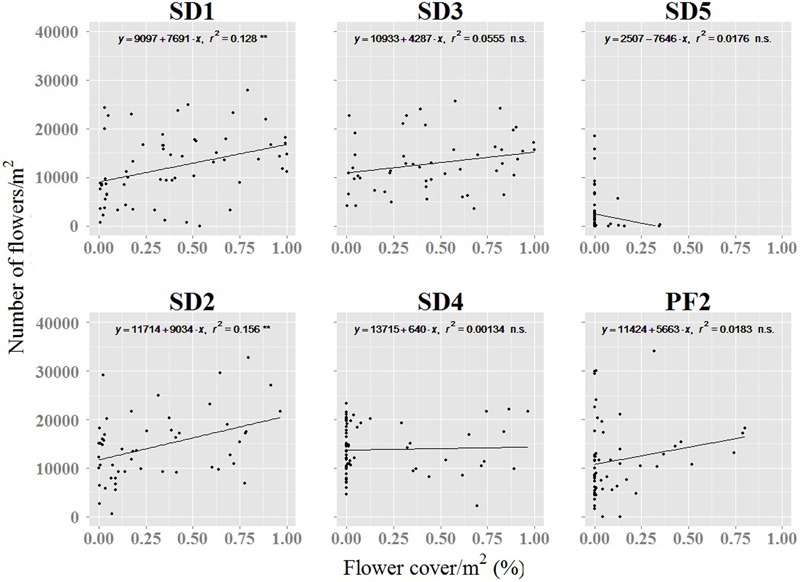
Relation between the numbers of flowers counted in the field and the percentage of flower cover detected by UAV. ^∗∗^*p* < 0.01; ^∗∗∗^*p* < 0.001; n.s., not significant. PF1 was not included because field sampling was not performed for this site. Name of study sites as in **Figure 2**.

The relationship between the number of flowers quantified during the fieldwork and the percentage of flower cover detected by the UAV was weak (**Figure 7**). The highest values were found in the primary and stabilized dunes where the flights were performed at the peak of flowering (SD1 and SD2, **Figure 7**). Lowest and non-significant correlations were observed in those areas where UAV flights occurred after the peak of flowering (SD4 and SD5) or where *A. longifolia* invades the understory of pine forest (PF2, **Figure 7**).

## Discussion

### Detection of *A. longifolia* Flower Distribution and Cover

Our results using pixel-based Random Forest classification confirm the ability of using UAV to map invasive plants since we obtained very high classification accuracy in all our experimental locations. These results with the Random Forest algorithm are in-line with other works in the field of Remote Sensing (Immitzer et al., 2012; Feng et al., 2015; Tamouridou et al., 2016) and with object-based classification (Michez et al., 2016a; Yuan and Hu, 2016). This was expected due to the unique bright yellow appearance of *A. longifolia* flowers in the winter season in juxtaposition to the lack of yellow flowering in most co-occurring native or exotic plants (Flora-On: Flora de Portugal Interactiva, 2014).

The use of UAV in the field of invasive alien plants has recently become more common (Hill et al., 2016; Müllerová et al., 2017a). This technology can be a major contribution to monitor any phenomena that induces changes on plant phenology or morphology such as the spread of a forest pest (Lehmann et al., 2015b) or the establishment of a biocontrol agent. In our study, *T. acaciaelongifoliae* expansion is expected to reduce flowering (i.e., decrease quantity of flowers through space and time as it spreads from the release points) and vigor of *A. longifolia* (Dennill, 1988), and as such a high resolution Remote Sensing protocol to monitor the plant flowering may be an excellent solution to be used as a proxy to monitor temporal and spatial impact of this biocontrol agent.

Additionally, and despite a detailed cost analysis was not performed, the costs of monitoring the flowering in the field versus the costs of just deploying the UAV and processing the image were clearly different: flower monitoring at each field site (which sampled some plots and not the entire area overflow by the UAV) required four researchers to work for about 8 h while each UAV flight was done in less than 2 h with 2 operators (at some locations 1).

In studied sand dunes, as well as in other dune systems (Elorza et al., 2004; Rascher et al., 2011), *A. longifolia* frequently replaces native plant communities where shrubs and herbs are more frequent, occupying large patchy areas and becoming dominant as an overstory species, and so is easier to detect. As expected (Perroy et al., 2017), when *A. longifolia* occurs in the understory of the pine forests, it is not detected by the UAV. Nevertheless, at the peak of flowering *A. longifolia* visibility increased in the more open areas of the pine forest canopy.

Accuracy of classification was dependent of flowering season and habitat type, showing better values during flowering peak and in sand dunes where *A. longifolia* dominates the overstory. Even so, when flowers were hidden by pine canopy or when UAV flights occurred at the end of flowering season and less or senescent (weak yellow coloring) flowers were present, highly accurate values were still obtained. Using UAV technology to map below the canopy remains a challenge (Perroy et al., 2017) but new developments of unmanned aerial systems are starting to address flights in forests understory (Brust and Strimbu, 2015; Liao et al., 2016; Michez et al., 2016b). These results highlight the potential of this approach to detect variations on *A. longifolia* flowering, confirming their plausible use as a proxy to detect the decrease in flower production due to the establishment of the biocontrol agent.

The flexibility in the acquisition of imagery with UAV allows capturing specific stages of phenology (Müllerová et al., 2017b); in our study it allowed to capture the peak of flowering for most of the study areas. We observed that shadows can have very negative effects on imagery captured at high latitudes during the days of clear-sky. This happens due to the low altitude of sun on the local horizon even during the solar noon. Acquisitions during full cloud coverage significantly reduced the impact of shadows in our experiment, and did not have a meaningful impact on the high accuracy of the image classification exercise.

In a broader perspective, the high accuracy in identifying *A. longifolia* flowering confirms the potential for using UAVs to access phenological changes due to biota attack or any other factor that induce changes in plant phenology or morphology. This complies with other studies, e.g., that used satellite imagery to assess the impact of pine beetles (Skakun et al., 2003) and confirms the importance of accurate evaluation of plant characteristics. Such studies highlight how UAV techniques can be flexible and applied in a wide range of study fields. Not only forestry, but also agriculture and environment, are increasingly evaluating Remote Sensing approaches for improving management efficiency (Wulder et al., 2006; Xiang and Tian, 2011; Burkart et al., 2014).

### Testing Correlation Between Flower Cover by UAV and Field Measured Flower Number

The variation of phenology and structure of *A. longifolia* added to the variability of the multiple habitats where the species occurs and this increased the challenge of quantifying the flowers by UAV-based imagery. *Acacia longifolia* has a high structural variation as it can develop flowers along its branches, at different heights and overlapping (or not) each other, as confirmed during field measurements: e.g., dense groups of *A. longifolia* trees with high numbers of branches and flowers overlapping each other several times vs. trees with dispersed flowers that rarely overlap; flowers at the top of the canopy which are hard to reach (small trees up to 8 m) vs. flowers at soil level that are easily quantified (shrubs lower than 2 m). This high level of variation occurred within the same trees as well as between different trees and was, most likely, the main factor that contributed to the low correlation found between the numbers of flowers measured in the field and those estimated using UAV imagery. Few works have tried to evaluate this type of relation.

Thorp and Dierig (2011) provided a good example of the application of this methodology achieving much better correlation for *Lesquerella fendleri* (*Brassicaceae*). However, *L. fendleri* is an herb/forb which may produce all the flowers at approximately the same level; additionally, their experiment was conducted in highly controlled conditions with only one unique area of 1 m^2^ being observed through time, which was not the case in our variable field conditions.

While our field measurements could potentially benefit from some re-design to better characterize what is in fact perceived by the UAV sensor and the inherent variability of *A. longifolia* flowering which is probably the main cause of the observed lack of correlation. To address what is detected by the UAV we suggest that the quadrats should be marked on the field prior to the UAV flights to ensure that positioning errors from GNSS receiver are not responsible for the mismatch. Furthermore, the volume of *A. longifolia* should be also considered by establishing allometric equations relating the volume with above ground biomass (Cunliffe et al., 2016).

This research was one of the first attempts to quantify the number of flowers using UAVs and although this goal was not satisfactorily achieved, it brought valuable insights on the limitations and potentials of these systems. The quantification of flowers, fruits, galls and/or any other plant trait is of increasing importance to Remote Sensing of vegetation and thus, the use of low-cost UAV as well as new satellite observation platforms (e.g., Sentinel) deserve further exploration.

## Conclusion

A successful low-cost approach was developed to map *A. longifolia* flowering using UAV. Mapping of *A. longifolia* flowering is the first step on establishing a method for monitoring the effect of the biocontrol agent *T. acaciaelongifoliae*. And while we failed to establish a predictive relationship between area of flower cover and number of flowers, we believe that this method can be used for monitoring the expected decrease of the production of flowers.

Despite the challenges and limitations, our results showed that UAVs clearly offer a simple and reliable method to map the distribution of the invasive *A. longifolia*. Even if this approach is better suited when *A. longifolia* is in the peak of flowering and occurs as an overstory species. While in the future it is expect that the biocontrol will significantly diminish the flowering intensity, this impact will increase gradually over time, therefore the near post-flowering period reflects the expected immediate impacts of the biocontrol.

*Acacia longifolia* flower cover was not linearly correlated with the number of flowers, oppositely to *L. fendler*i, indicating that there is a need to develop better methods to estimate *A. longifolia* flowering. This lack of correlation is likely explained by variability of the structure and variability of *A. longifolia* flowering. Considering the potential advantages that may come from the improvement of this approach in field conditions, future works should address these challenges and limitations opening a broader field of applications.

## Author Contributions

All the authors participated during the fieldwork for the acquisition of flower counts as well as in the writing of thispublication. The main writer was NdS while SC and PC assisted specially in the sections of Remote Sensing and methodology. EM, HM, and FL-N assisted in the writing of all sections regarding with *A. longifolia* and its biological control as well the study area description and methodology. The image processing task of this publication were performed by NdS.

## Conflict of Interest Statement

The authors declare that the research was conducted in the absence of any commercial or financial relationships that could be construed as a potential conflict of interest.

## Footnotes

1 www.firemap.pt

2 www.sensefly.com

## Supplementary Material

FIGURE S1 Random Forest supervised classification results for SD1 and SD2.

FIGURE S2 Random Forest supervised classification results for SD3.

FIGURE S3 Random Forest supervised classification results for SD4 and SD5.

FIGURE S4 Random Forest supervised classification results for PF1 and PF2.

FIGURE S5 Flower cover per m2 for SD1 and SD2.

FIGURE S6 Flower cover per m2 for SD3.

FIGURE S7 Flower cover per m2 for SD4 and SD5.

FIGURE S8 Flower cover per m2 for PF1 and PF2.

FIGURE S9 Example of flower visibility during the flowering **(a)** and fructification **(b)** seasons. Areas dominated by *A. longifolia* can be visually identified during both seasons, but are more clearly detected during the flowering period.

FIGURE S10 Variable importance as identified by the supervised Random Forest classification. Blue band was generally the most important variable for the classifier which can be interpreted as a result of yellow (high red/green vs. low blue color).
