# Supplementary figures and images for: Mapping the Flowering of an Invasive Plant Using Unmanned Aerial Vehicles: Is There Potential for Biocontrol Monitoring?
Source: Front Plant Sci. 2018 Mar 8;9:293. doi: 10.3389/fpls.2018.00293 (PMC5853265; doi:10.3389/fpls.2018.00293)

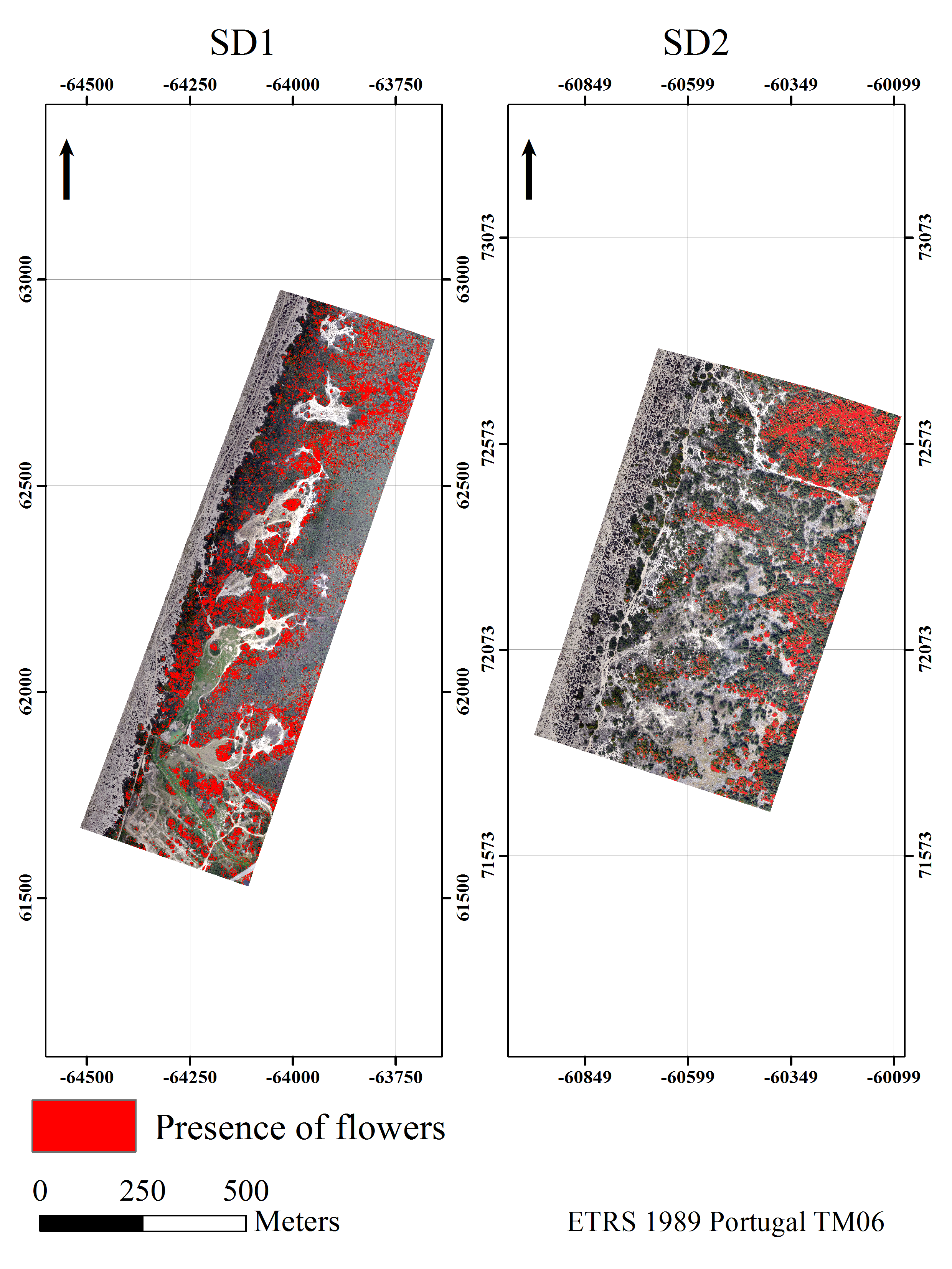

Supplement: FIGURE S1 — Random Forest supervised classification results for SD1 and SD2. [file Image_1.TIF]

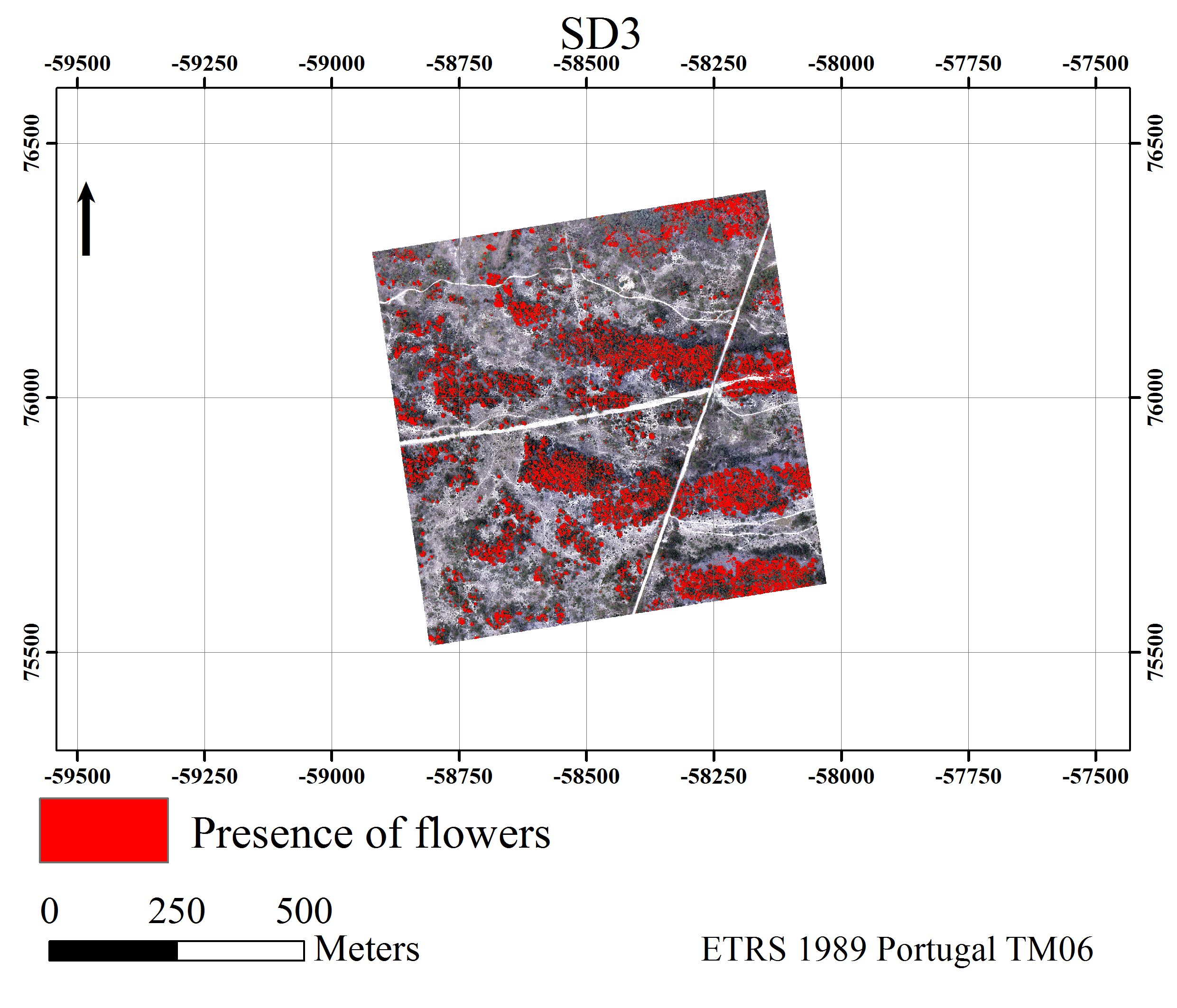

Supplement: FIGURE S2 — Random Forest supervised classification results for SD3. [file Image_2.TIF]

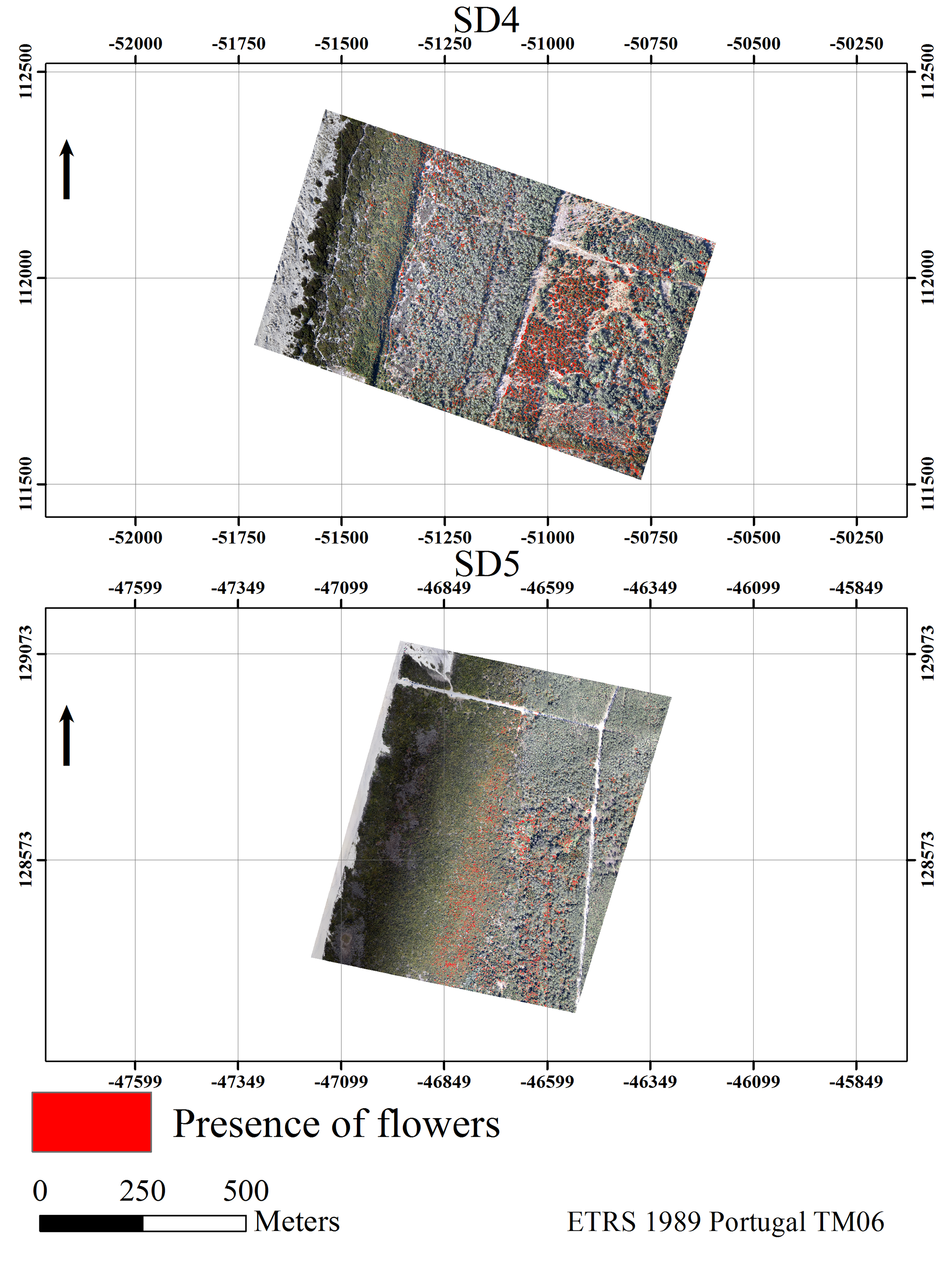

Supplement: FIGURE S3 — Random Forest supervised classification results for SD4 and SD5. [file Image_3.TIF]

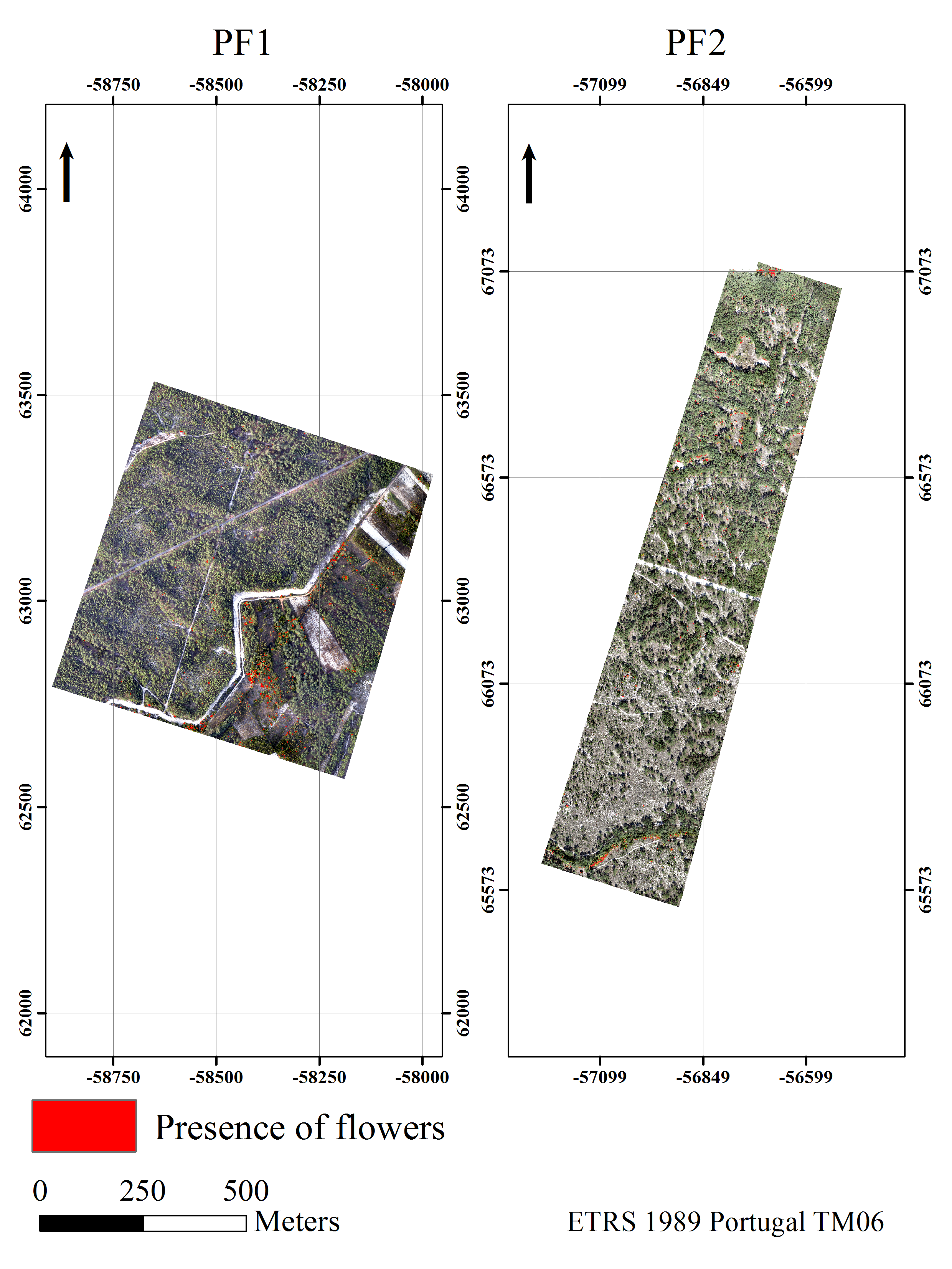

Supplement: FIGURE S4 — Random Forest supervised classification results for PF1 and PF2. [file Image_4.TIF]

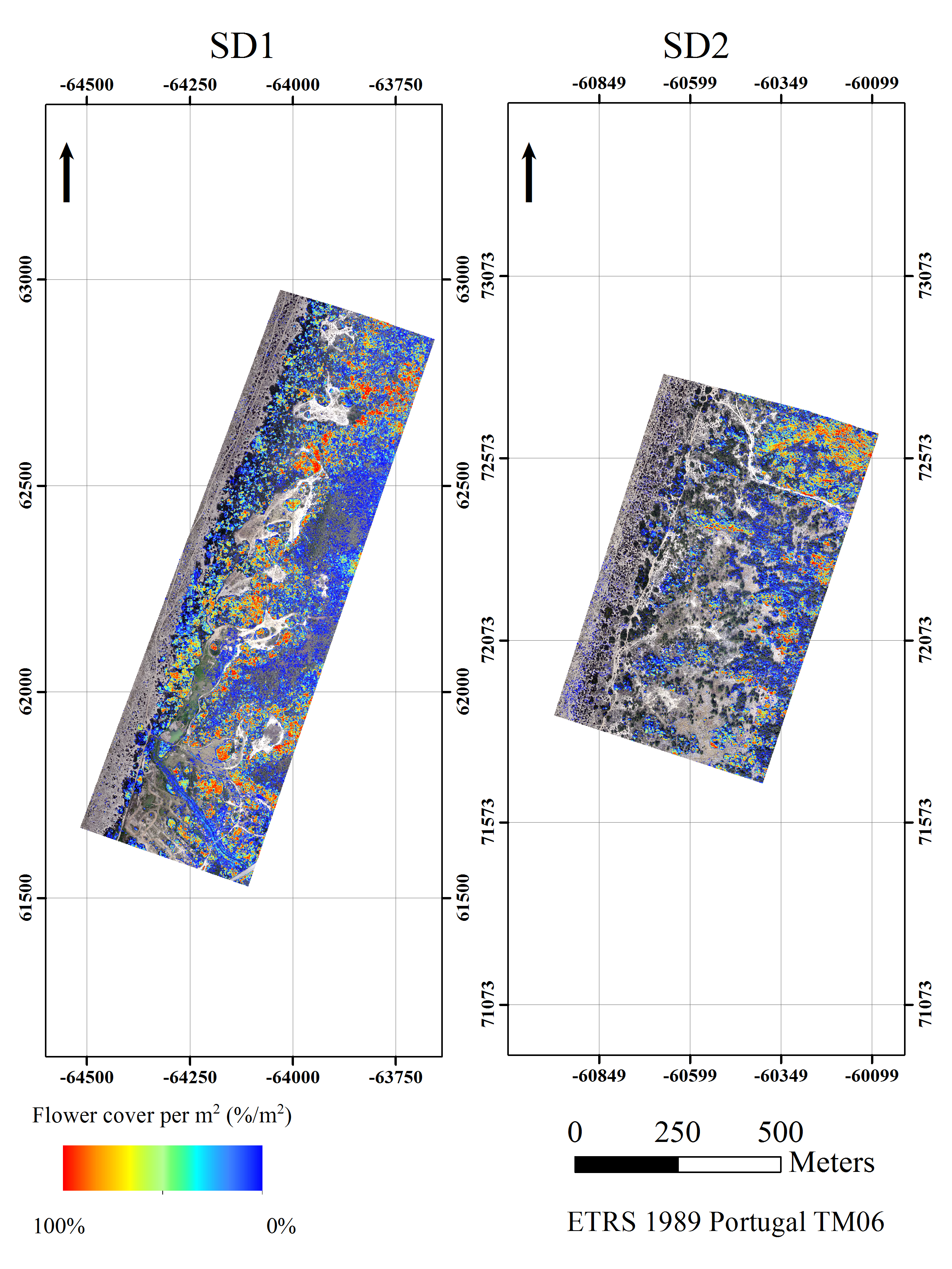

Supplement: FIGURE S5 — Flower cover per m2 for SD1 and SD2. [file Image_5.TIF]

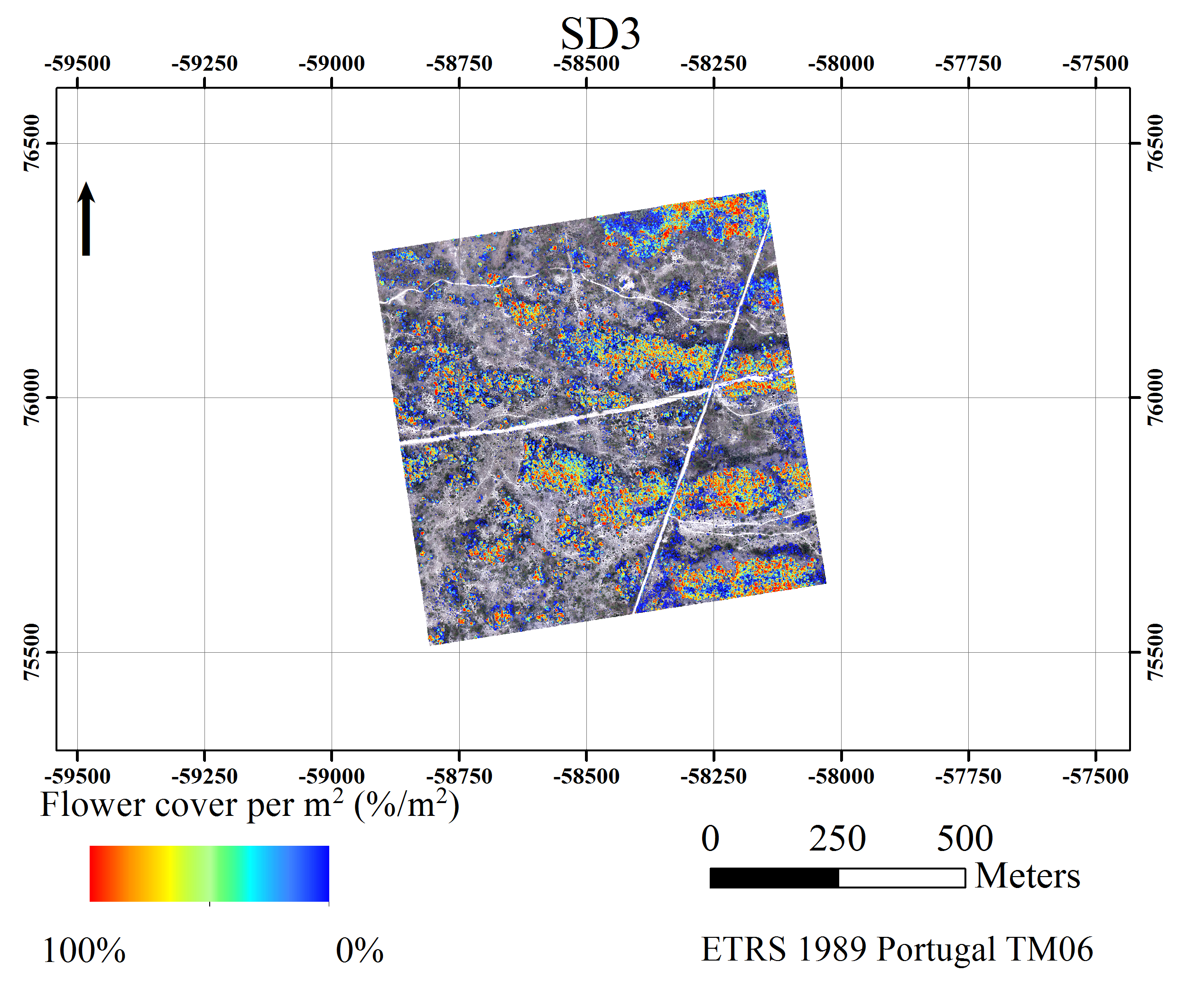

Supplement: FIGURE S6 — Flower cover per m2 for SD3. [file Image_6.TIF]

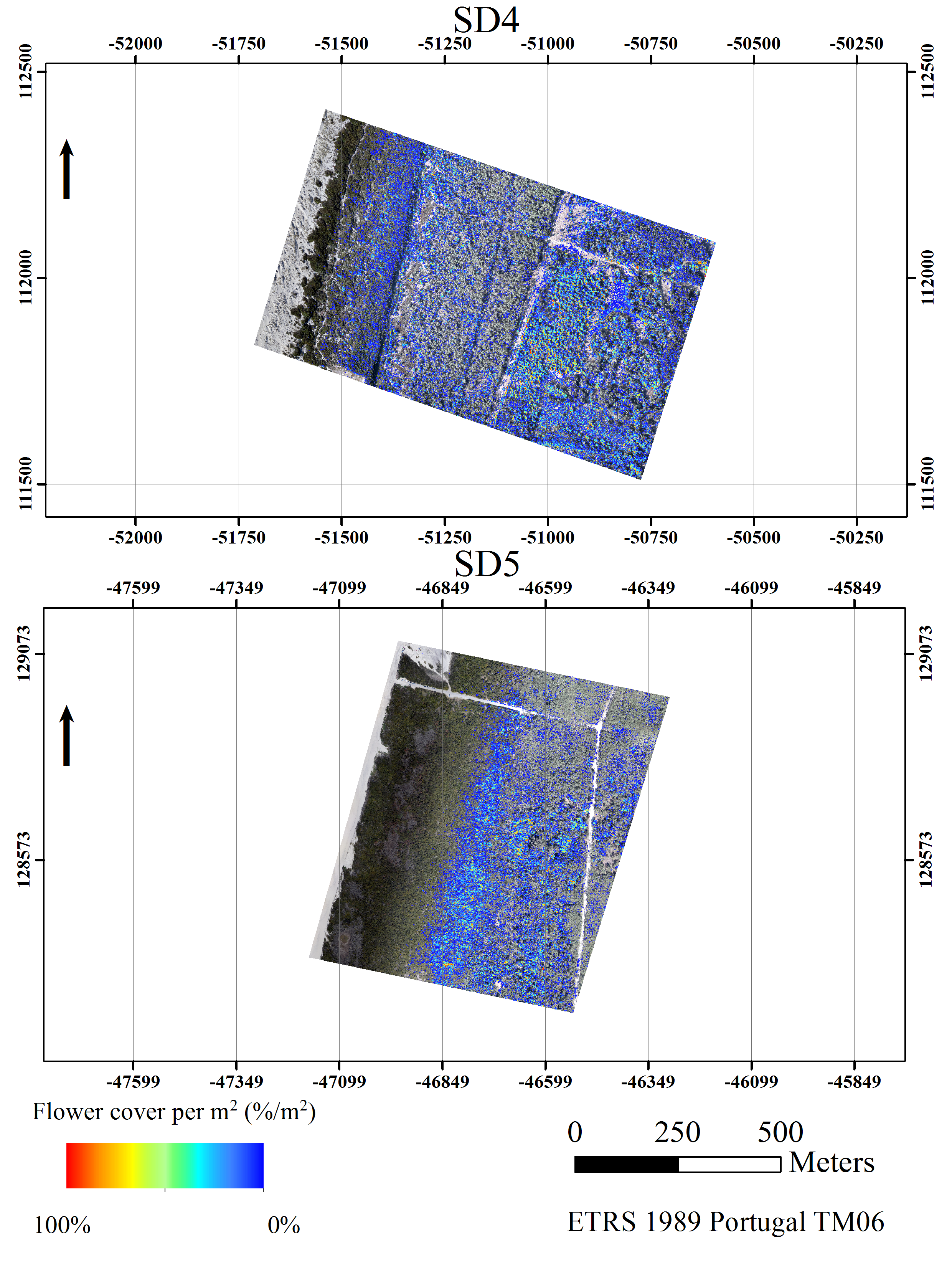

Supplement: FIGURE S7 — Flower cover per m2 for SD4 and SD5. [file Image_7.TIF]

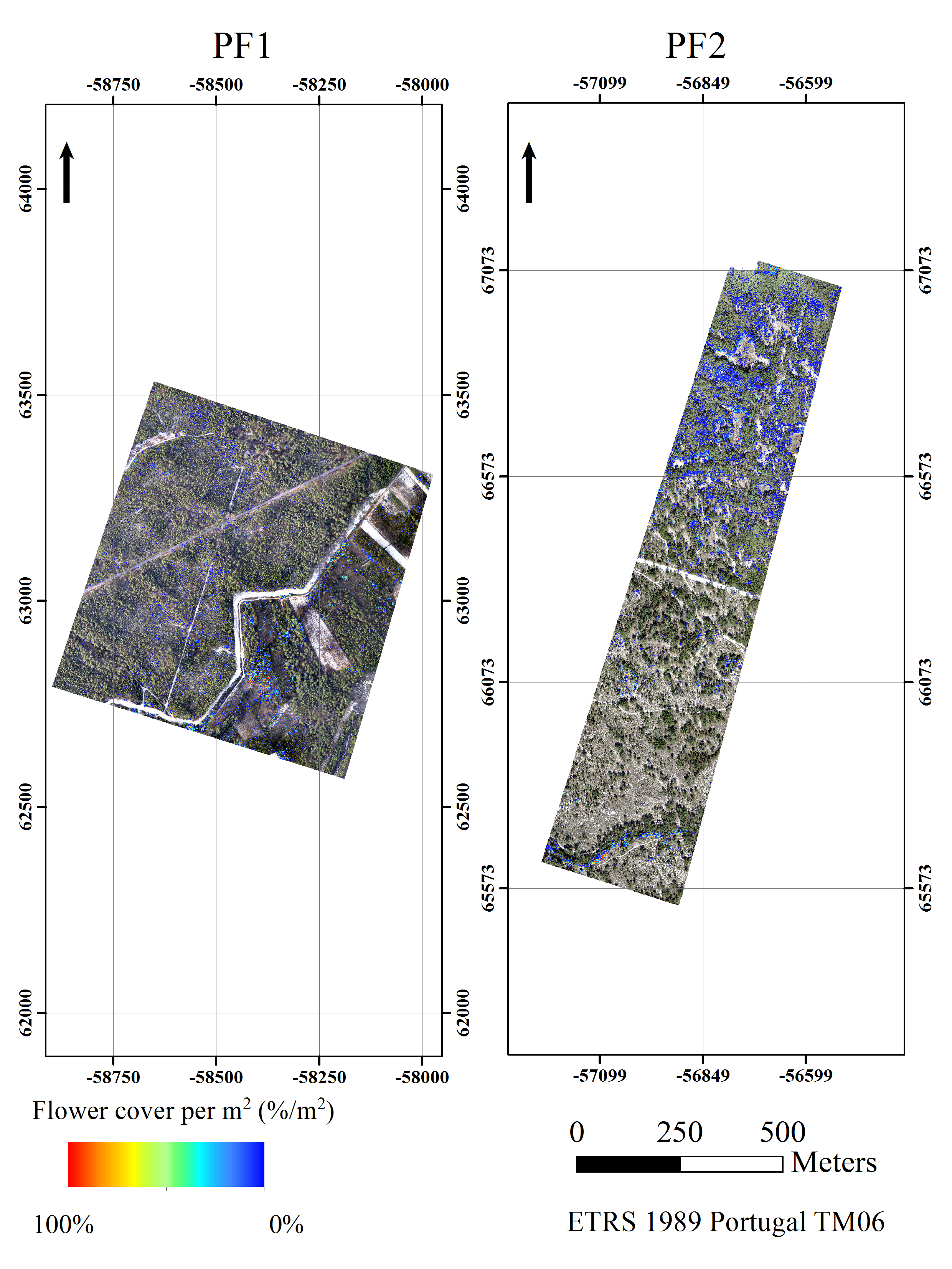

Supplement: FIGURE S8 — Flower cover per m2 for PF1 and PF2. [file Image_8.TIF]

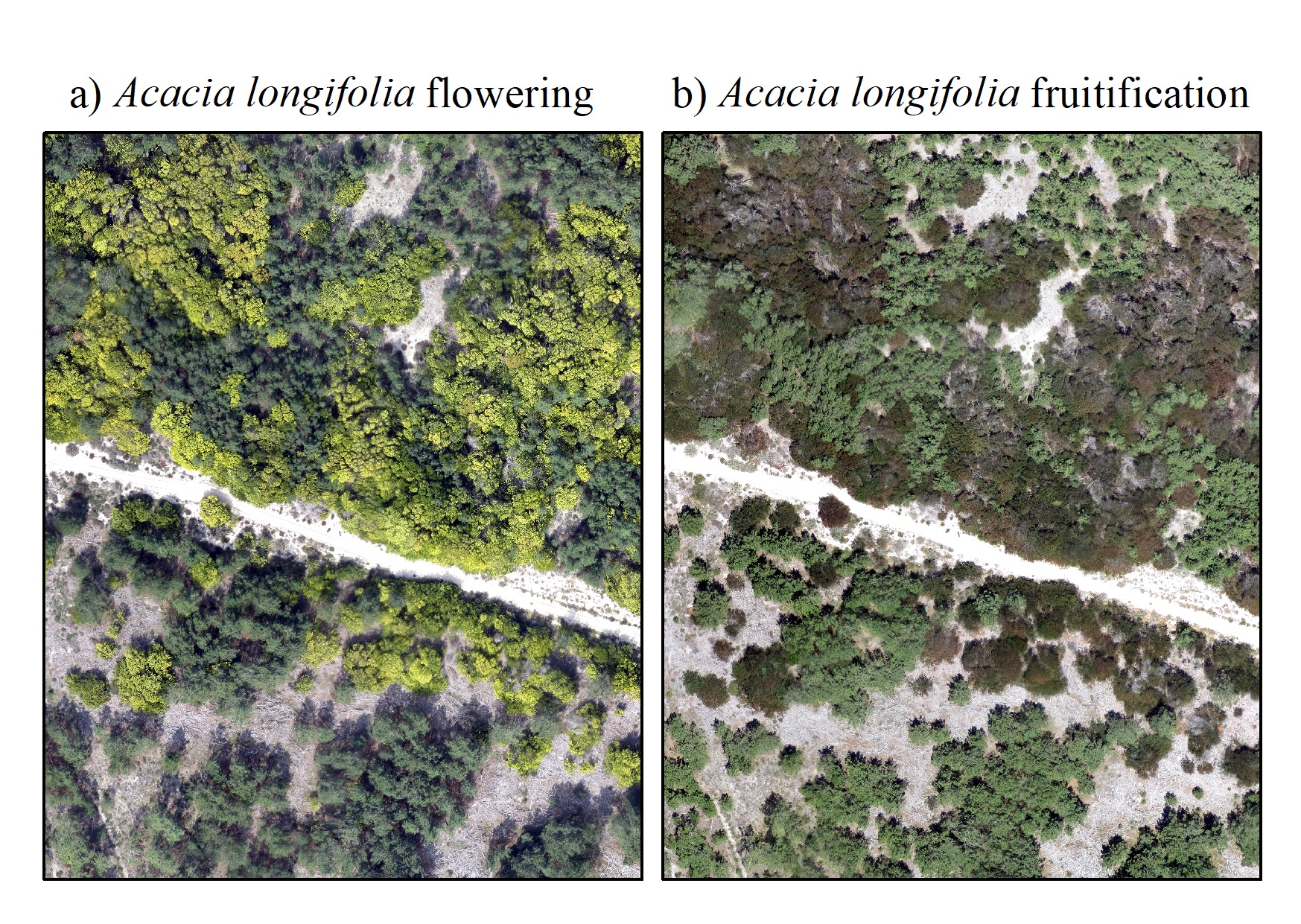

Supplement: FIGURE S9 — Example of flower visibility during the flowering (a) and fructification (b) seasons. Areas dominated by A. longifolia can be visually identified during both seasons, but are more clearly detected during the flowering period. [file Image_9.JPEG]

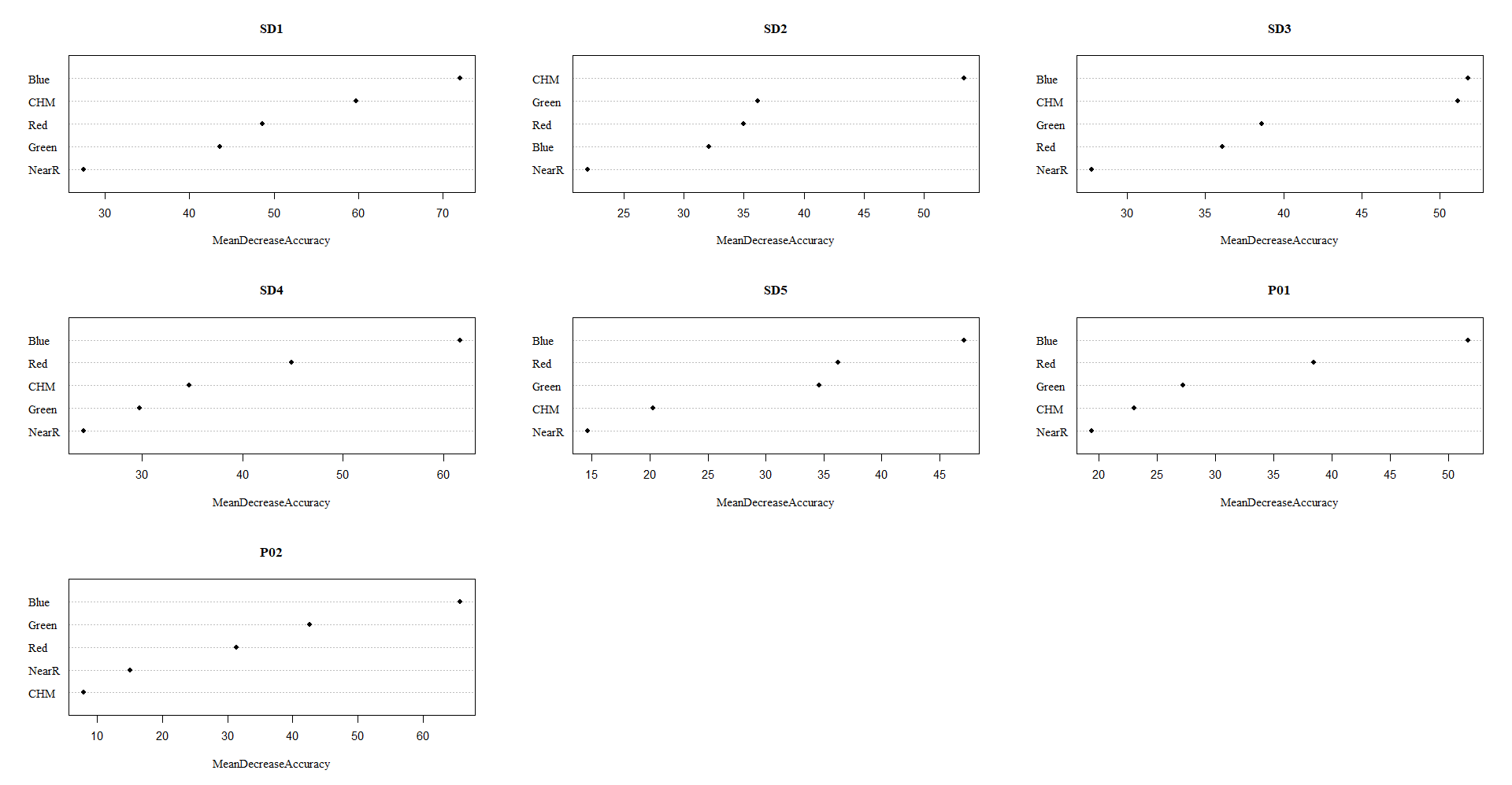

Supplement: FIGURE S10 — Variable importance as identified by the supervised Random Forest classification. Blue band was generally the most important variable for the classifier which can be interpreted as a result of yellow (high red/green vs. low blue color). [file Image_10.PNG]
